# Improved Thermal Stability of a Novel Acidophilic Phytase

**DOI:** 10.4014/jmb.2311.11044

**Published:** 2024-04-03

**Authors:** Byung Sam Son, So Hyeong Kim, Hye-Young Sagong, Su Rin Lee, Eun Jung Choi

**Affiliations:** Institute of Biotechnology, CJ CheilJedang Co., Suwon 16495, Republic of Korea

**Keywords:** Acidophilic, dual optimum pH, phytase, phytic acid, thermostable

## Abstract

Phytase increases the availability of phosphate and trace elements by hydrolyzing the phosphomonoester bond in phytate present in animal feed. It is also an important enzyme from an environmental perspective because it not only promotes the growth of livestocks but also prevents phosphorus contamination released into the environment. Here we present a novel phytase derived from *Turicimonas muris*, TmPhy, which has distinctive structure and properties compared to other previously known phytases. TmPhy gene expressed in the *Pichia* system was confirmed to be 41 kDa in size and was used in purified form to evaluate optimal conditions for maximum activity. TmPhy has a dual optimum pH at pH3 and pH6.8 and exhibited the highest activity at 70°C. However, the heat tolerance of the wildtype was not satisfactory for feed application. Therefore, random mutation, disulfide bond introduction, and N-terminal mutation were performed to improve the thermostability of the TmPhy. Random mutation resulted in TmPhyM with about 45% improvement in stability at 60°C. Through further improvements, a total of three mutants were screened and their heat tolerance was evaluated. As a result, we obtained TmPhyMD1 with 46.5% residual activity, TmPhyMD2 with 74.1%, and TmPhyMD3 with 66.8% at 80°C heat treatment without significant loss of or with increased activity.

## Introduction

Phosphorus is an essential macro mineral along with calcium for the growth of monogastric animals. Feed ingredients are mostly plant-derived and contain 50-80% of the total phosphorus in the form of phytic acid. If animals can effectively digest the phytate-bound phosphorus in feedstuff, then no or only little supplementation of expensive inorganic phosphate will be needed allowing for maximum nutritional value to be obtained with minimal resource input [1]. As interest in environmental phosphorus pollution issues increases, laws are being enacted to reduce phosphorus losses from pig and poultry farming facilities. Non-ruminant animals do not have sufficient phytase activity in the stomach and small intestines, which causes a large amount of phosphorus in the feed to be unused and excreted hence causing environmental pollution [2]. Therefore, there has been a continuous need to supply phytase, an enzyme that decomposes phytic acid, to livestock along with feed, and to discover novel phytases which can be used for feed application.

As phytic acid passes through the intestine (alkaline conditions), it combines with minerals such as zinc, calcium , and iron to form compounds, which makes intestinal digestion difficult [3]. As a result, phytase must decompose phytic acid as much as possible in the animal’s stomach (pH 2-4 conditions). For this reason, a variety of acidophilic phytases have been reported, including rPhyPt4 from *Acidobacteriaceae* bacterium S15 showing activity of 2,790 μmol/min/mg at 37°C, pH 2.5 condition and *Acidobacteria* derived rPhyA with an activity of 1,792 μmol/min/mg [4, 5]. Both enzymes have the same optimal pH of 2.5 and are expected to be suitable for use as feed enzymes. Unfortunately, very few acidophilic phytases have been identified so far, and most commercial phytases currently used, from the following origins (*E. coli*, *Citrobacter braakii*, *Aspergillus niger* etc.) have optimal pH at slightly acidic (pH 4~6) conditions [6].

Additionally, to facilitate the use of enzymes as feed additives, the development of highly active phytase is necessary. Phytase has been classified into four types according to its structure. ① Histidine acid phosphatase (HAP), ② ß-propeller phytase, ③ cysteine phosphatase, and ④ purple acid phosphatase [7]. Among them, HAP is known to decompose phytic acid most efficiently due to its enzymatic property of cleaving most of the 6 phosphates on phytate. This is the main reason that all phytases used as animal feed supplement today belong to the HAP family of phytases [8].

Furthermore, heat-resistance is also a prerequisite for enzymes for feed application because feed usually go through the pelleting process, in which they are treated with high-temperature steam and get exposed to friction heat damage during the formation of pellet through the die [9]. To resolve the issue of thermal labile properties of feed enzymes, three different methods are normally used. First is coating the enzyme with fat, mineral or polymers, second is to discover naturally thermostable enzymes, and third is to genetically manipulate the enzyme to obtain intrinsic thermal stability. Encapsulation of the enzyme is a method that has been successfully used in many commercial feed enzymes, but coating can have a negative effect on the availability of the enzyme after ingestion. It can delay the release of the enzyme and cause reduction in the speed of activation of the enzyme, which is especially important for phytase because of the need for exogenous phytase to immediately act on phytate in the acidic stomach of the animal [10]. Thus, the second and the third route was used to increase the thermal stability of the phytase in this study. A number of phytases including *A. niger* PhyB (60°C), *A. ficcum* PhyB (65°C), and *Schwanniomyces castellii* phytase (77°C) have been reported to have an optimal temperature of 45-70°C [11], and we have found a phytase originating from *Turicimonas muris* (TmPhy) which has similar optimal temperature. The development of phytase that can withstand high temperature environment is pivotal for expanding the uses of enzymes. Using TmPhy as the starting enzyme, we used protein engineering methods to increase its thermal stability. This study sought to discover and develop a phytase with high activity under acidic condition with heat stability.

## Materials and Methods

### Chemicals and Reagents

Phytic acid, ammonium molybdate, nitric acid and ammonium metavanadate were purchased from Sigma-Aldrich. KOD polymerase was purchased from TOYOBO, Japan. The pH buffers were sourced from Biosesang. Reagents for purification were from Qiagen, Netherland.

### Construction of the Expression Plasmid

The novel phytase gene sequence was obtained from the NCBI (National Center for Biotechnology Information) under the Accession No. WP_277039767.1 and the codon was optimized for *Pichia* expression by the gene synthesis company Cosmogenetech. Sequence alignment with the sequence in the PDB, 1DKQ, was performed to remove the signal peptide, and the gene was amplified by PCR using 5’-AGAGGCTGAAGCTCA AGAATTGATCCCAGGAACTCTG-3’ and 5’-TGATGATGCTCGAGATCATTAGCAATACATCTAGAATCCAAT-3 primers. The amplified TmPhy gene was cloned into pPICZa (Invitrogen, USA) using an infusion cloning kit (Takara, Japan) and the recombinant plasmid was cleaved into linear form by SacI treatment.

### Protein Expression and Purification in *P. pastoris*

For expression of the TmPhy gene, *Pichia pastoris* bg10 (BioGrammatics, USA) was used. Competent cells were prepared and linearized recombinant plasmid was introduced into *P. pastoris* according to the method of Kumar, R. [12]. Primers (5’-ACAGCACAAATAACGGGTTATTGTTTATAAAT-3’ and 5’- AATGATTTTCCC AAACCCCTACCACAAGATATTC-3’) were utilized for the screening of transformants and the selected transformants were pre-cultured in 1% BMGY medium for TmPhy expression. Pre-culture was carried out at 28°C for 48 h at 220 rpm and cells were recovered by centrifugation. The recovered cells were seeded into the main culture (0.1% BMGY) and incubated at 220 rpm for 24 h at 28°C. The culture medium was supplemented with 2%methanol, which was added every 24 h. After 4 days of main culture, cells and supernatants were separated by centrifugation, and the separated supernatants were buffer exchanged with lysis buffer using an Amicon® Ultra-15 Centrifugal Filter Unit (Merck, Germany).

Recombinant proteins in lysis buffer were purified by Ni-NTA chromatography method. The prepared protein was dispensed onto a column filled with Ni-NTA beads and allowed to adsorb onto the beads. Then, wash buffer (20mM imidazole) was used to remove unwanted proteins, and finally elution buffer (250mM inidazole) was run to obtain only the desired proteins.

### Assay of Phytase Activity

To evaluate phytase activity of enzymes, a reaction solution of 2 mM phytic acid and 250 mM glycine/HCl pH3 buffer was previously kept at 37°C in a thermomixer (Eppendorf, EP5382000023). An appropriate amount of purified enzyme was mixed into the reaction solution and the reaction was stopped with chromogenic buffer after a certain amount of time. To prepare the chromogenic buffer, 65-68% nitric acid, 100 g/l of ammonium molybdate and 2.35 g/l of ammonium metavanadate were mixed at 2:1:1. The absorbance was measured at 415 nm. One unit (U) of the phytase activity is defined as the amount of enzyme that releases 1 μmol of Pi per minute.

### Characterization of Recombinant Phytase

For the evaluation of optimal conditions, phytase activity was measured with 2 mM phytic acid and 250 mM buffer. To determine the effect of pH on activity, glycine/HCl buffer was used for pH 2.5-3, acetate buffer for pH 4-5, and Tris-HCl buffer for pH 6-10. In addition, the effect of metal on the enzyme was evaluated in the presence of 1 mM metal ions. Evaluation of both the optimal pH and the effect of metal were performed at 37°C. The optimal temperature was evaluated at each temperature in glycine/HCl pH3 buffer, and thermostability was determined by placing the purified enzyme on ice after 5 min heat treatment at each temperature and then checking the residual activity in glycine/HCl buffer pH3 at 37°C. For enzyme kinetics, phytase activity was estimated over a range of substrate concentration (0-2.5mM) using 0.1 U/ml of purified enzyme at 37°C, pH 5.5 and kinetic constants were calculated from a Lineweaver-Burk plot.

### Mutant

**Library screening and disulfide bond introduction**

To enhance the heat resistance of TmPhy, we made an error prone library using the TmPhy gene as the template. Taq polymerase (SolGent) was used to amplify the TmPhy gene, and the PCR conditions were adjusted to a concentration of 0.04 mM manganese, and D_2_O was added to reach a total volume of 20 ul [13]. The amplified gene was constructed into a recombinant plasmid as in method 1 and introduced into *P. pastoris*. To screen for superior transformants, the culture broth (1% BMGY 5ml) was supplemented with 2% methanol, which was added every 24 h. After 48 hours of induction, the supernatant was allowed to stand at 70°C for 1 min and the residual activity was measured. As a result, TmPhyM mutants with better thermal stability than the wildtype was screened (data not shown). The disulfide bond candidates were derived from the model structure of TmPhyM obtained from a template-based structure modeling protocol GalaxyTBM [14]. To introduce disulfide bonds (Table S1) into the TmPhyM gene, site directed mutagenesis was performed according to the method of Kunkel, T. A. [15]. To obtain N-terminal deletion mutant, we used the TmPhyDM1 gene as the template and the following primer pairs, 5'-AGAGGCTGAAGCTTGCGAGTTGGAAAAAGTTGTTGTTGT-3' (TmPhyDM2) and 5'-TGATGATGATGCTCGAGATCATTAGCAATACATCTAGAATCCAATC-3' for PCR. For N-terminal random mutation of TmPhyDM1, we used the same template and downward primer as above and a degenerate oligonucleotide 5'-AGAGGCTGAAGCTNNKNNKNNKNNKNNKNNKNNKNNKNNKNNKGTTTATTGCG AGTTGGAAAAAGTTGTTG-. 3' for the upward primer. PCR was carried out and the products were used to select the most thermotolerant mutant of TmPhyMD1, TmPhyMD2 and TmPhyMD3. The process then proceeded according to the error prone library screening method.

## Results and Discussion

### Temperature and pH Profiles of TmPhy

Phytase is the primary enzyme used in feed applications worldwide, and efforts have been made to identify novel phytases from many sources. A novel phytase from an anaerobic bacteria, *Turicimonas muris*, was identified. A comparison of the differences between TmPhy and five well-studied representative phytases from *Escherichia coli* (PDB ID: 1DKM), *Citrobacter braakii* (3ZHC), *Klebsiella* sp. ASR1(2WU0), *Aspergillus fumigatus* (1QWO), and *Aspergillus niger* (3K4P) showed similar structural features of the HAP family of phytases but only 39%, 34%, 28%, 19%, and 18% sequence similarity, respectively, indicating that TmPhy shares very low sequence similarity with previously identified phytases [16-20]. In addition, TmPhy was 367 amino acids long, as opposed to the 410-450 amino acid residues of expressed form of most phytases. It was expected that this difference might cause it to exhibit different features from other phytases. For the expression of TmPhy, it was cloned into a pre-prepared vector [21] and expressed in *P. pastoris*. Purified TmPhy was obtained from the fermentation broth and the enzyme was characterized.

**Fig. 1:**
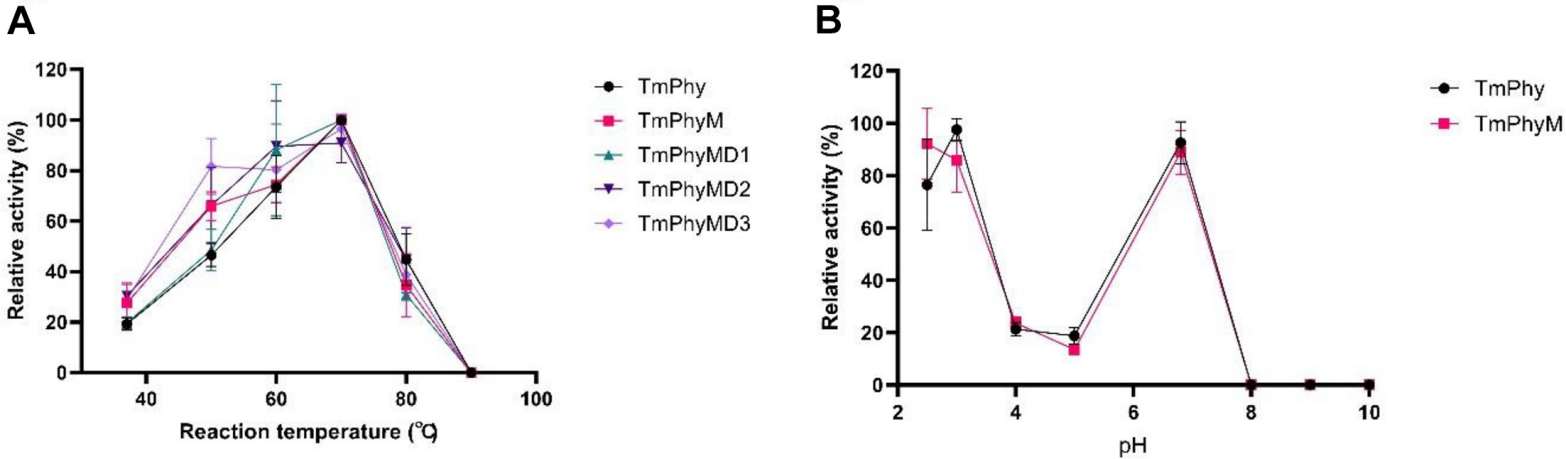
Temperature profile (A) and pH profile (B) of TmPhy and mutants.

The temperature profile of TmPhy was measured at pH3 and showed the highest activity at 70°C, with approximately 5.2-fold increase in activity compared to 37°C (Fig. 1A). A number of phytases including *A. niger* PhyB (60°C), *A. ficcum* PhyB (65°C), and *Schwanniomyces castellii* phytase(77°C) have been reported to have an optimal temperature of 45-70°C [11], and TmPhy was found to have a similar optimal temperature. The optimal pH profile of TmPhy showed a dual optimum pH, with high activity at pH 2.5-3, decreasing at pH 4-5, and increasing again at pH 6.8 (Fig. 1B). Other phytases with more than one optimal pH have priorly been reported [22-24]. TmPhy showed high activity at pH 2.5-3, which opens up expectation for high digestibility through gastrointestinal action during animal feeding. In addition, TmPhy has specific activity of 4322.5 ± 40.8 U/mg at 37°C (Table 1), which is very high compared to commercially available wildtype enzymes (811-1800 U/mg for *E. coli* phytase and 3457 U/mg for *C. braakii* derived phytase) [6]. Collectively, due to the high activity at low pH, TmPhy is expected to have superior phytic acid degradation ability in the stomach. This could lower the production of metal ions-phytate compounds which forms due to the increase in pH that occurs during the digestion process in the small intestine and could be expected to have nutritional benefits.

### Effects of Metal Ions on the Activity of Purified TmPhy

Feedstuffs contain various amino acids, starch, non-starch polysaccharides, fats, vitamins and minerals, and are known to contain small amounts of metal ions [25, 26]. Phytate is anionic in nature and forms complex compounds with divalent cations in alkaline conditions, which are insoluble and hinder absorption in the gastrointestinal tract, reducing the bioavailability of minerals [27]. Therefore, phytase activity less affected by metal ions and high activity in acidic conditions are crucial characteristics required for an effective phytase. The effect of metal ions on TmPhy is shown in Table 3. TmPhy is not significantly affected by the different types of metal ion. In addition, the activity increased slightly with the addition of all ions, except for nickel. This suggests that small amounts of metal ions in the feed are expected to support the activity of TmPhy.

### Evaluation of the Specific Activity and Heat Tolerance of TmPhy Mutants

The pH, temperature, and metal properties of TmPhy were sufficient for its use as a feed enzyme. However, the heat resistance evaluation of TmPhy showed that the residual activity at 70°C was about 43.6%, and the sample lost all activity when heat treated at 80°C. Therefore, we designed an experiment to improve the thermal stability of TmPhy. Attempts to increase thermal stability have been studied in various ways, including glycosylation optimization [28], domain shuffling [29], and spycatcher system [30]. In this study, we aimed to improve thermal stability by utilizing error prone library screening and introducing disulfide bonds. First, we acquired a number of thermal stable modifications using multiple rounds of error prone library screening. Primary screening by thermal stability increase resulted in a double mutant K49P/G58Y which showed 10% presidual activity increase under the screening selection criteria compared to the wildtype TmPhy. Consecutive rounds of screening using the best mutant as the template resulted in the addition of three mutation (M33T/E44D/G62A) with 25%p increase in thermal stability. The final selected additional mutant, T63Q, ultimately resulted in a seven-residue mutant (M33T/E44D/E48P/K49P/G58Y/G62A/T63Q) TmPhyM, compared to the wildtype sequence. Purified TmPhyM showed a 44% improvement in residual activity at 60°C over TmPhy (Fig. 3). Four of the seven mutations were changes to residues on helices. Two Glys on helices are mutated which is predicted to stabilize the protein based on the fact that Gly with additional degrees of rotation stabilizes the unfolded state rather than folded secondary structures [31] . Two Pros are added to a loop which might decrease the floppiness of the loop and increase the stability of the protein [32].

**Fig. 2:**
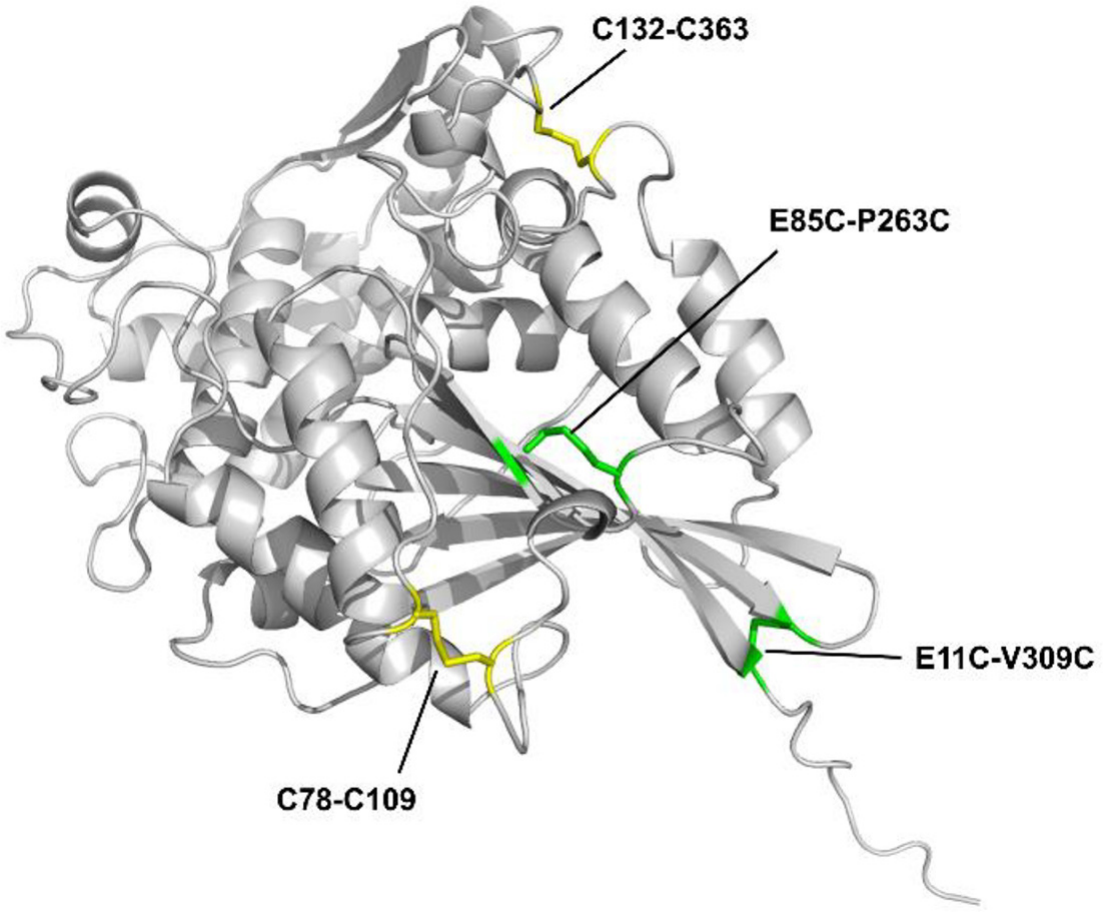
Natural and engineered disulfide bonds in the TmPhyMD1 model structure. Yellow shows the disulfide bonds of the wildtype, and green shows the additional disulfide bonds that were introduced to increase thermal stability.

**Fig. 3:**
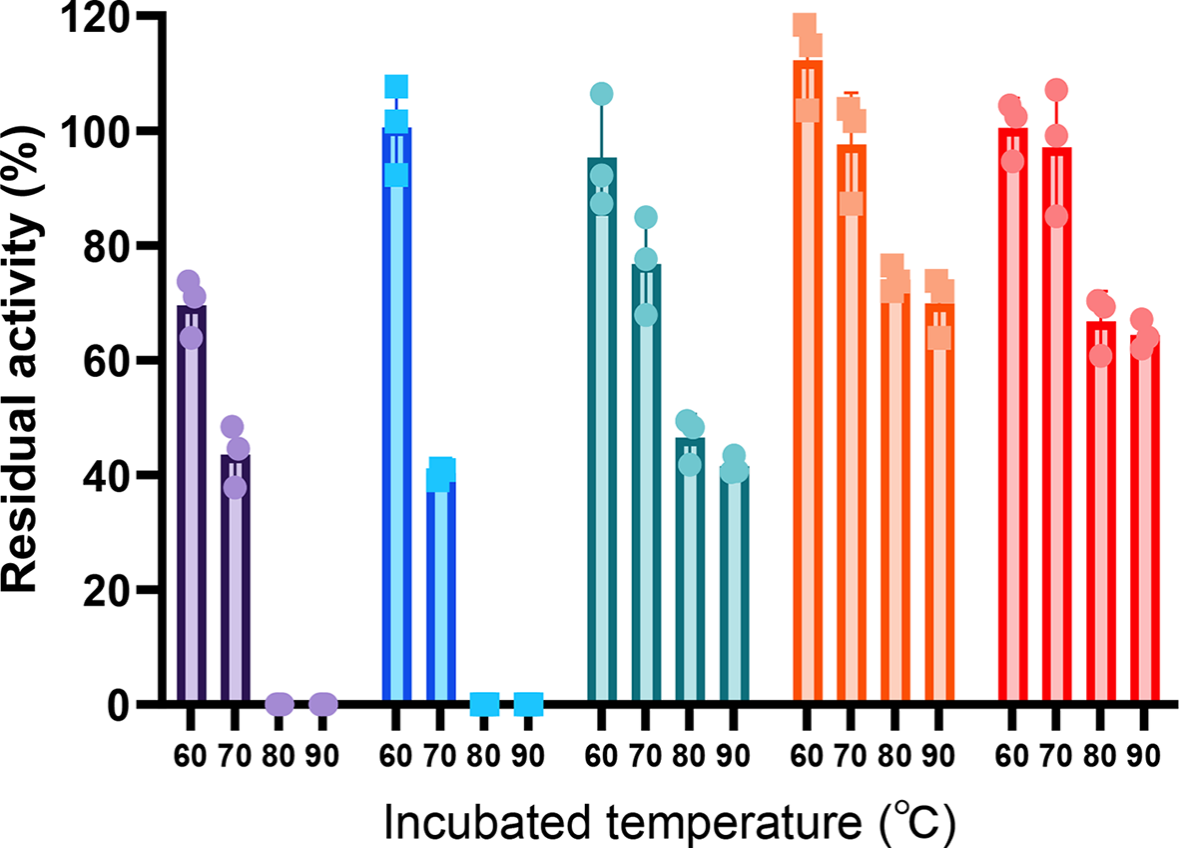
Thermostability of the TmPhy and mutants. The bars in the graph are colored as follows. TmPhy is in purple, TmPhyM in blue, TmPhyMD1 in green, TmPhyMD2 in orange and TmPhyMD3 in red.

The introduction of disulfide bonds into TmPhyM was considered to further improve thermal resistance. Disulfide bonds have been used to improve thermal stability in a variety of enzymes, including endoglucanases [33] , lipases [34, 35], xylanases [36], and also phytases [37]. To select the residues to be substituted with cysteine, we explored a list of residues with Cα-Cα distance between 3.0-7.5 Å [38], of which 31 pairs of single or combined disulfide bond modifications were constructed and evaluated (Table S1). Out of the 12 single disulfide bonds tested, significant thermal stability improvement was seen in the disulfide bond modification containing the substitution E11C/V309C, where at 80°C residual activity increased from null to over 30%. Additional 9 disulfide bonds were added to E11C/V309C and evaluated, resulting in TmPhyMD1(E11C/V309C/E85C/P263C) with even higher increase in thermal stability of 46% residual activity at 90°C (Table S1). Addition of a third disulfide did not increase the thermal stability beyond TmPhyMD1. The dramatic thermal stability increase of TmPhyMD1 comes with a price however because the specific activity decreased 3.4 times than that of TmPhyM (Table 1). To determine the stabilizing effects of the two disulfide bonds engineered in TmPhyMD1 independent of the other mutations, we placed the disulfides onto the wildtype enzyme producing TmPhy-E11C/V309C, TmPhy-E85C/P263C and TmPhy-E11C/V309C/E85C/P263C. Residual activities at different incubation temperatures were evaluated (Table S2). E11C/V309C and E85C/P263C both improved the thermal stability of the wildtype protein with the largest contribution by E11C/V309C. Combining the two disulfide bonds (TmPhy-E11C/V309C/E85C/P263C) resulted in additive thermal stability increase. The location of the two designed disulfide bond and the two natural disulfide bonds which exist in the wildtype enzyme is shown in Fig. 2.

N-terminal modifications were made to TmPhyMD1 based on several reports which suggest that the N-terminal region may affect stability [39]. In particular, in [Bei, 2009], domain shuffling of *A. niger* phytase and *A. fumigatus* phytase was performed to evaluate the stability of each site, and it was found that when the N-terminal domain of *A. niger* phytase was replaced with that of *A. fumigatus* phytase, the thermal stability increased [40]. Based on this, we tested two N-terminus engineering methods on TmPhyMD1, N-terminus deletion and N-terminal substitution. From the first experiment we acquired TmPhyMD2, found to have 10 amino acids deleted at the N-terminus. From the second engineering method we acquired TmPhyMD3, found to have 8 amino acids, LNSSVPGA, substitution at the N-terminus. TmPhyMD2 and TmPhyMD3, which were further modified from TmPhyMD1, were found to have improvement in both specific activity and heat resistance compared to TmPhyM. TmPhyMD2 retained about 70% residual activity up to 90°C, and both N-terminus modifications showed 1.4-fold higher specific activity than TmPhyM. This confirmed that the N-terminal region not only plays an important role in thermal stability, but also affects activity. Temperature, pH profile and kinetic parameters for the wildtype enzyme and the mutant in this study is shown in Fig. 1 and Table 2.

## Conclusion

In conclusion, we aimed to develop a new generation of feed grade phytase with outstanding characteristics. TmPhy and its mutants exhibited higher activity under acidic conditions, and heat tolerance was improved by the introduction of multiple mutations through various methods. For further use as an industrial feed enzyme, it is necessary to select an appropriate expression system to produce the developed enzyme and optimize the expression conditions. *Bacillus* [41], *Pichia* [42, 43], *Trichoderma* [44] are commonly used hosts for protein expression, and the next task is to introduce these superior variants into the appropriate host and evaluate their expression.

## Figures and Tables

**Table 1:** Specific activity of TmPhy and mutants.

Enzyme	Specific activity (U/mg)
TmPhy	4322.5 ± 40.8
TmPhyM	6684.9 ± 144.4
TmPhyMD1	1958.6 ± 12.9
TmPhyMD2	9983.5 ± 213
TmPhyMD3	9361.3 ± 675.2

The phytase activity was measured at pH3 and 37°C.

**Table 2:** Kinetic parameters for TmPhy and mutants.

	Km (mM)	kcat (s-1)	kcat/Km (s-1mM-1)
TmPhy	0.56 ± 0.05	112.4 ± 11.1	200.5 ± 19.8
TmPhyM	0.30 ± 0.03	115.9 ± 6.5	391.4 ± 22.1
TmPhyMD1	0.32 ± 0.05	3.8 ± 0.4	12.0 ± 1.2
TmPhyMD2	0.28 ± 0.02	205.6 ± 9.0	742.9 ± 32.7
TmPhyMD3	0.36 ± 0.02	80.0 ± 3.2	221.7 ± 8.9

All parameters were measured at pH5.5 and 37°C.

**Table 3:** Cation effects on TmPhy activity.

Metal	ion Relative activity(%)
Control	100.0 ± 14.9
Ca^2+^	110.8 ± 10.8
Mg^2+^	125.3 ± 17.3
Mn^2+^	123.4 ± 12.1
Zn^2+^	106.9 ± 10.2
Ni^2+^	99.8 ± 7.5
Cu^2+^	115.8 ± 27.9
Co^2+^	113.5 ± 18.0

The phytase activity was measured at pH3 and 37°C with 1 mM each cation.
